# Nitrate-containing beetroot juice reduces oxygen consumption during submaximal exercise in low but not high aerobically fit male runners

**DOI:** 10.20463/jenb.2016.0029

**Published:** 2016-12-31

**Authors:** Colin R. Carriker, Roger A. Vaughan, Trisha A. VanDusseldorp, Kelly E. Johnson, Nicholas M. Beltz, James J. McCormick, Nathan H. Cole, Ann L. Gibson

**Affiliations:** 1 Department of Exercise Science, High Point University, High Point U.S.A; 2 Department of Exercise Science and Sport Management, Kennesaw State University, Kennesaw U.S.A; 3 Department of Health, Exercise and Sports Sciences, University of New Mexico, Albuquerque U.S.A

**Keywords:** Nitric Oxide, Dietary Nitrate, Plasma Nitrite, Aerobic Fitness, Exercise Nutrition Physiology, Submaximal Oxygen Cost

## Abstract

**[Purpose]:**

To examine the effect of a 4-day NO_3_^-^ loading protocol on the submaximal oxygen cost of both low fit and high fit participants at five different exercise intensities.

**[Methods]:**

Eleven (6 high fit, VO_2max_ 60.1 ± 4.6ml/kg/min; 5 low fit, VO_2max_ 42.4 ± 3.2ml/ kg/min) participants were initially assigned to a placebo (PL; negligible NO_3_^-^) or inorganic nitrate-rich (NR; 6.2 mmol nitrate/day) group using a double-blind, placebo-controlled, crossover design. Participants completed three trials (T1, T2 and T3). T1 included a maximal aerobic capacity (VO_2max_) treadmill test. A 6-day washout, minimizing nitrate consumption, preceded T2. Each of the four days prior to T2 and T3, participants consumed either PL or NR with the final dose 2.5 hours prior to exercise. A 14-day washout followed T2. T2 and T3 consisted of 5-minute submaximal treadmill bouts (45, 60, 70, 80 and 85% VO_2max_) determined during T1.

**[Results]:**

Low fit nitrate-supplemented participants consumed less oxygen (p<0.05) at lower workloads (45% and 60% VO_2max_) compared to placebo trials; changes were not observed in high fit participants. The two lowest intensity workloads of 45 and 60% VO_2max_ revealed the greatest correlation (r=0.54, p=0.09 and r=0.79, p<0.05; respectively) between VO_2max_ and change in oxygen consumption. No differences were found between conditions for heart rate, respiratory exchange ratio or rating of perceived exertion for either fitness group.

**[Conclusion]:**

Nitrate consumption promotes reduced oxygen consumption at lower exercise intensities in low fit, but not high fit males. Lesser fit individuals may receive greater benefit than higher fit participants exercising at intensities <60% VO_2max_.

## INTRODUCTION

Foods containing dietary nitrate (NO_3_^-^) have recently emerged as a potential ergogenic aid for endurance athletes^1^^–^^3^. Dietary inorganic nitrate can be found in a variety of foods such as root and green leafy vegetables, as well as some cured/processed meats^4^. Additionally, acute supplementation can elevate concentrations of plasma nitrate and nitrite ([NO_3_^-^] and [NO_2_^-^]; respectively) as early as two and a half hours post-ingestion. Like vegetable food sources, supplementation with sodium nitrate has also been shown to elevate both [NO_3_^-^] and [NO_2_^-^] in as little as two days of supplementation^5^. Dietary NO_3_^-^ may enhance athletic performance^6^^-^^8^ via the rise in [NO_2_^-^], a biomarker of increased nitric oxide (NO) availability^9^^,^^10^. Interestingly, NO_3_^-^ supplementation has been reported to reduce oxygen consumption at pre-established submaximal exercise intensities, which suggests dietary nitrate improves oxygen utilization^5^^,^^7^^,^^11^. Despite this evidence linking NO_3_^-^ supplementation with reduced oxygen cost, a recreationally trained cohort appear to be those receiving the greatest benefit (VO_2max_ 40-60 ml/ kg/min)^1^^,^^3^^,^^7^. In contrast, many of the studies examining well-trained or highly fit participants (VO_2max_ > 60 ml/kg/min) supplementing with nitrate found no change in submaximal oxygen cost and no improvement in performance compared to placebo conditions^12^^–^^14^. Together, as alluded to by others, these data suggest dietary and supplemental NO_3_^-^ affect athletic performance to a magnitude largely influenced by training status^15^^,^^16^. The purpose of this study was to examine the effect of a 4-day NO_3_^-^ loading protocol on the submaximal oxygen cost of both low fit and high fit participants at five different exercise intensities. We hypothesized that increasing [NO_2_^-^] via dietary NO_3_^-^ supplementation would reduce oxygen consumption at 45, 60, 70, 80, and 85% VO_2max_ in low but not high fit participants. We also hypothesized that NO_3_^-^ supplementation would alter oxygen consumption in a manner dependent on aerobic fitness, with low fit participants experiencing the greatest increase in metabolic efficiency.

This study was conducted with treadmill exercise while other studies have examined the effects of nitrate via cycling protocols. Cycling and running exercise are inherently different in terms of muscle recruitment which creates difficulty with direct comparison of results. This study also differs from previous literature by examining the changes in oxygen consumption in both high and low fit participants following identical supplementation protocols. It is difficult to compare results of other studies with either high or low fit cohorts as supplementation protocols may not be similar. While other studies have, in large part, examined a single workload, this study examined 5 workloads to better understand the efficacy of dietary nitrate supplementation over a range of exercise intensity relative to each participant.

**Table 1.** Participant descriptive data.

Participants	Group	Age (yr)	HT (cm)	WT (kg)	BMI	VO_2max_ (ml/kg/min)
n = 5	Low fit	23 ± 3	178.02 ± 6.02	80.53 ± 19.18	25.30 ± 5.25	42.4 ± 3.2
n = 6	High fit	24 ± 4	182.03 ± 4.30	80.21 ± 9.64	24.18 ± 2.54	60.1 ± 4.6

Values are means ± SD. Height, HT (cm); weight, WT (kg); body mass index, BMI (kg/m^2^); maximal oxygen consumption, VO_2max_ (ml/kg/min).

## METHODS

Eleven healthy men (6 high fit with VO_2max_: 60.1±4.6 ml/kg/min and age 23±3; 5 low fit with VO_2max_: 42.4±3.2 ml/kg/min and age 24±4; Table 1) provided written consent for this study as approved by the University of New Mexico’s institutional review board (#13-288). Participants were excluded if they indicated any known cardiovascular, pulmonary or metabolic disease on a health history questionnaire. Participants were classified with either high or low aerobic fitness based on their VO_2max_ (>85% or <65% of age and male gender norms; respectively) according to previously published fitness categories for maximal aerobic power^17^. Participants who achieved a VO_2max_ between 65 and 85% of age and male gender norms were excluded from the study to better create heterogeneity between high and low fit groups.

**Figure 1.**
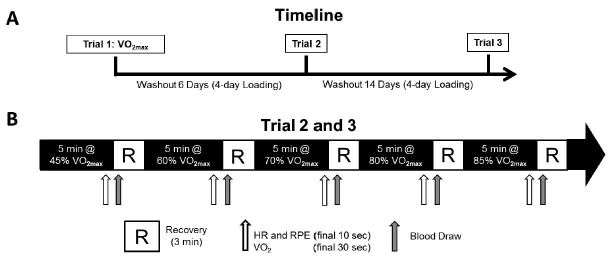
Trial Timeline Summary. A) Schematic timeline: Trial 1 (VO_2max_ test) and Trial 2+3 (either placebo or nitrate-rich supplementation). B) Trial 2 and 3 submaximal exercise (45, 60, 70, 80, and 85% of V · O2max). Laboratory resides at ~1600m.

All tests were conducted on a treadmill with the velocity and grade controlled manually (Precor C966i, Woodinville, WA). During trial 1 (T1), all participants completed a standardized VO_2max_ treadmill test to exhaustion; the protocol varied for participants prescreened as fit or unfit based on self-reported weekly physical activity. Following an initial 2-minute slow walk at 1% (1.34 and 1.79 m/s for unfit and fit; respectively) the velocity increased by 0.67 m/s every minute and a half for the first 6 stages. Treadmill grade then increased by 3% every 1.5 min. Breath-by-breath metabolic gases were continuously collected (True One, ParvoMedics, Sandy, Utah, USA) and averaged over 11-breath sequences. The VO_2max_ was recorded as the highest value collected over the 30 seconds prior to the participant’s volitional exhaustion. Oxygen consumption data were then plotted against treadmill velocity during the first 6 stages (grade at 1%) to establish the velocity corresponding to 5 different intensities (45, 60, 70, 80, and 85% VO_2max_) to be used during trial 2 (T2) and trial 3 (T3). During T2 and T3, metabolic gasses were analyzed as described above to assess VO2 over the final 30 seconds of each five-minute submaximal exercise bout. Heart rate (HR) was measured via short range radiotelemetry (Polar Electro T31, New York, USA). HR and rating of perceived exertion (RPE; 6-20 scale)^18^ were recorded during the final 10 seconds of each exercise bout. Figure 1 depicts the testing sequence including washout and supplement loading periods between trials as well as T2 and T3 details.

Following T1, in a randomized, double-blind, placebo- controlled, crossover fashion, participants were initially assigned to a placebo (PL) or nitrate-rich (NR) beverage group. Each participant received four 70ml beverages (either four placebo or four NR) and instructions to consume one beverage per day following the designated washout period and prior to T2. The opposite treatment beverage was consumed prior to T3. Beverages were consumed at approximately the same time of day with the final dose consumed 2.5 hours prior to commencing exercise. The PL beverage was black-currant juice with negligible NO_3_^-^ content^1^^, ^^6^^, ^^11^ and the NR trial consisted of a commercially available, inorganic nitrate-rich product, (Beet It with 6.2 mmol NO_3_^-^, James White Drinks Ltd, Ipswich, UK). Beverages were similar in color and were provided to participants in identical packaging (bottles) to minimize indicators of treatment (PL or NR). During the dietary nitrate washout periods (Figure 1), participants were asked to minimize their consumption of nitrate-rich foods and supplements containing nitrate/l-arginine^4^ based on a list of items provided to each participant.

Participants were asked to avoid strenuous exercise, alcohol, chewing gum, and mouthwash 24 hours prior to each trial and caffeine 12 hours before each trial as such items may attenuate the rise in [NO_2_^-^] following dietary NO_3_^-^ supplementation^19^. Participants self-recorded their dietary intake prior to T2 and then consumed the same diet prior to T3.

Upon arrival to the lab for T2 and T3, an intravenous catheter (Venflon IV cannula; Becton-Dickinson, Franklin Lakes, New Jersey, USA) was placed in a prominent forearm vein. Blood samples (~10 ml) were drawn and collected into a tube containing lithium heparin before exercise and following each intensity (45%, 60%, 70%, 80%, and 85% of VO_2max_) for analysis of [NO_2_^-^] during both T2 and T3 (Figure 1B). Prior to each blood draw, ~3 ml sample was drawn and discarded to clear the line of saline. A 5 ml saline flush was administered following the 10 ml blood draw. The catheter was removed following the post-85% VO_2max_ blood draw.

All tubes were inverted approximately ten times and immediately centrifuged at 4,000 rpm at 4°C for 6 minutes. Plasma samples were then stored at -80°C for subsequent analysis. Plasma nitrite was measured in duplicate using a commercially available microplate-based colorimetric assay kit (Cayman Chemical, USA). The inter- assay coefficient of variation was within the range as indicated by the manufacturer.

Results are presented as mean ± standard deviation. Data were analyzed using a two-way analysis of variances (ANOVA) to examine variables between condition (NR or PL) at each intensity (45%, 60%, 70%, 80%, and 85% of VO_2max_). Significant findings were determined by p < 0.05 using a Bonferioni post-hoc when an interaction effect was observed. The magnitude of linear association between oxygen consumption at a given intensity and VO_2max_ was measured with the Pearson’s coefficient of correlation.

## RESULTS

**Figure 2.**
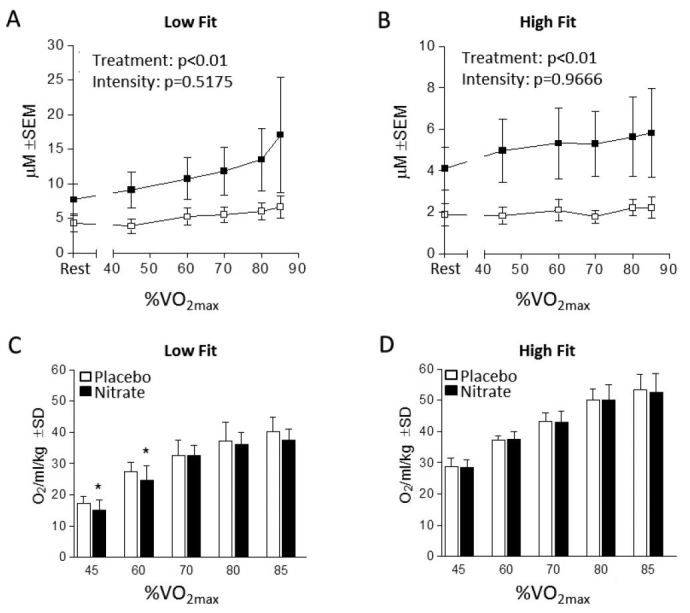
Plasma Nitrite and Submaximal Oxygen Costs. A) Plasma nitrite (μM) of low fit participants during submaximal exercise at baseline (Rest), 45%, 60%, 70%, 80% and 85% VO_2max_ after placebo (white) and nitrate-rich (black) supplementation. B) Plasma nitrite from high fit participants during the same submaximal exercise intensities described for panel A. C) Oxygen consumption of low fit participants (ml/kg/min) during submaximal exercise at 45%, 60%, 70%, 80% and 85% VO_2max_ after placebo (white bars) and nitrate-rich (black bars) supplementation 2.5 hours prior to exercise. D) Oxygen consumption of high fit participants (ml/kg/min) during the previously described submaximal exercise during intensities. ^*^Significance at p < 0.05.

We began our analyses by verifying that dietary NO_3_^-^ increased [NO_2_^-^]. As expected, [NO_2_^-^] was consistently greater in the NR group in both low fit and high fit participants (Figure 2A and 2B, respectively). Next, we measured the effect of dietary NO_3_^-^ supplementation on oxygen cost and observed that low fit participants exhibited a significantly reduced oxygen cost at 45% and 60% VO_2max_ (Figure 2C). Conversely, there were no differences between the NR and PL trials for the high fit participants (Figure 2D). High and low fit groups exhibited no differences in RER values between treatment (NR and placebo) at all intensities (Figure 4C and D).

**Figure 3.**
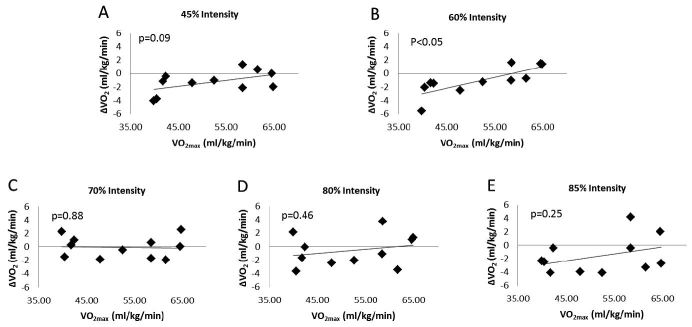
Aerobic fitness and change in VO_2_ following nitrate supplementation. Panels represent the correlation between participants’ aerobic fitness and change in VO_2_ following nitrate supplementation at the intensities indicated. A) 45% Intensity, r=0.54, p=0.09 B) 60% Intensity, r=0.79, p<0.05 C) 70% Intensity, r=-0.05, p=.88 D) 80% Intensity, r=0.25, p=0.46 E) 85% Intensity, r=0.38, p=0.25

In addition, the lowest intensity workloads of 45 and 60% revealed the greatest correlation (r=0.54, p=0.09 and r=0.79, p<0.05; respectively) between aerobic fitness and the change in oxygen consumption following nitrate consumption, suggesting NR beverages may possess greatest benefits at moderately intense exercise regardless of aerobic fitness (Figure 3).

**Figure 4.**
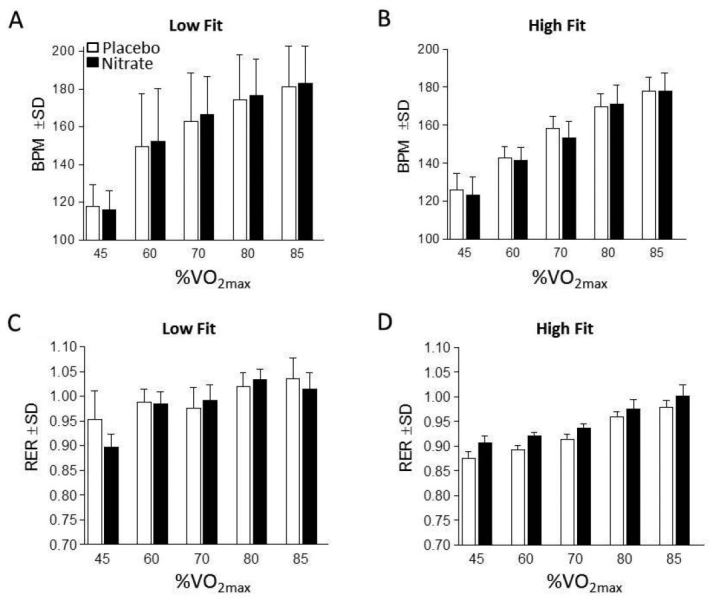
Submaximal Exertion Response A) Heart rate (HR) of low fit participants in beats per minute (BPM) during submaximal exercise at 45%, 60%, 70%, 80% and 85% VO_2max_ after placebo (white bars) and nitrate-rich (black bars) supplementation 2.5 hours prior to exercise. B) Participant HR of high fit participants during submaximal exercise during intensities described in panel A. C) Respiratory exchange ratio (RER). D) Participant RER of high fit participants during submaximal exercise during intensities described in panel A.

High and low fit participants’ HR responses were similar between their respective NR and placebo trials at all intensities (Figure 4A and B). There were also no differences in HR responses for any submaximal bout during the placebo and NR trials based on aerobic fitness comparisons. Likewise, RPE increased in an exercise intensity-dependent manner, but was more pronounced for the low fit participants. Regardless, no differences in perceived exertion between NR and PL trials at any of the five submaximal intensities were found for the low and high fit groups (data not shown).

## DISCUSSION

This study is one of the first to investigate the influence of aerobic fitness (VO_2max_) level (<65% or >85% normative values for men) in which participants completed the same supplementation protocol and exercised at 5 different submaximal intensities ranging from 45% to 85% of each participant’s respective VO_2max_. While our loading protocol (Figure 1A) was successful in elevating [NO_2_^-^] levels, the varying degree to which it did highlights the heterogeneity of our subject pool and may have precluded our ability to report statistically significant differences for oxygen cost reductions across all submaximal intensities.

The four-day loading protocol concluded with the final beverage ingested 2.5 hour prior to the first bout of submaximal exercise. Supplementation within two to three hours of exercise^6^^–^^8^ after a loading period as short as two^5^ or three^3^ days has been shown effective in increasing [NO_2_^-^]. As expected, we observed increases in the average [NO_2_^-^] values at baseline and throughout the NR trials as compared to PL for both our high fit and low fit participants (Figure 2A and 2B). As others have reported, the decrease in the O_2_ cost of exercise is negatively correlated with VO_2max_, such that aerobically fit individuals may experience less reduction in oxygen cost compared to a lesser fit cohort. In addition, the increase in NO_2_^-^ plasma levels following dietary NO_3_^-^ supplementation has been found to be lower in subjects with high VO_2max_. Both of these findings are consistent with our data^20^^,^^21^. Dietary nitrate supplementation increases NO bioavailability via the nitrate-nitrite-nitric oxide pathway^9^^,^^10^ and may, therefore, improve performance^22^^–^^24^; perhaps most notably in populations with lower aerobic fitness as explained below.

The current study results support previous investigations reporting that individuals who are more aerobically fit (as evidenced by a higher VO_2max_) incur a lesser reduction in submaximal oxygen cost when compared to less fit individuals^15^^,^^20^. Porcelli et al.^20^ examined changes in oxygen cost at 80% of individual ventilatory thresholds (reported as moderate-intensity exercise), while the current study examined 5 different training intensities relative to individual VO_2max_ (ranging from 45 to 85% VO_2max_). Dietary NO_3_^-^ supplementation for our high fit participants (VO_2max_: 60.1±4.6 ml/kg/min) failed to produce a statistically significant reduction in oxygen cost at any of the submaximal intensities (Figure 2B). In addition, the findings of our study support studies that examined changes in participants classified as low fit or recreationally active (VO_2max_ between 40 and 60 ml/kg/ min). In lesser fit participants, others have also found a reduction in oxygen cost followed dietary nitrate supplementation^2^^,^^3^^,^^7^. Therefore, findings for our low fit group (VO_2max_: 42.4±3.2 ml/kg/min), were expected, although interestingly the significant reductions in oxygen cost only occurred during the lower intensity exercise bouts (45 and 60% VO_2max_). While oxygen consumption was reduced for our low fit cohort, no differences in RER were found between PL and NR treatments for either the high or low fit group. Given the nature of physical activity, RER may not reflect macronutrient oxidation as other factors such as hyperventilation and lactate buffering may contribute to non-metabolic-created CO2. Future studies may wish to examine blood lactate in conjunction with metabolic gases to better elucidate the changes in oxygen consumption and macronutrient catabolism.

In a meta-analysis examining the effect of nitrate supplementation on exercise performance in healthy individuals, Hoon et al.^15^ reported, “Qualitative analysis suggested that performance benefits are more often observed in inactive to recreationally active individuals and when a chronic loading of NO_3_^-^ over several days is undertaken.” Given the number of studies published recently in addition to our findings, it is becoming evident that the benefits of dietary NO_3_^-^ may be limited to those individuals who have not already received the benefit of chronic physiological adaptation to aerobic exercise training^15^^,^^16^. Dietary nitrate has been implicated in improved mitochondrial efficiency, which may provide some indication of the mechanism through which some individuals receive benefit^25^^–^^27^. Although speculative, dietary nitrate may alter blood flow distribution including vasodilation of non-working muscle vascular beds as dietary nitrate does not prioritize a site-specific vasodilation to muscles engaged in exercise. In fact, blood flow distribution may, therefore, be reduced to working muscles within the limits of maximal cardiac output. In this manner, reduced blood flow may reduce oxygen delivery and transport to those muscles which need it most. Future research should also examine the changes in mitochondrial content and efficiency following dietary NO_3_^-^ controlling for aerobic fitness to better ascertain the benefits of this supplement alongside natural adaptations in response to chronic aerobic exercise.

The efficacy of dietary NO_3_^-^ may also be dependent on the amount consumed daily. Previous research found 3 of 10 participants who consumed 4 mmol NO_3_^-^ 2.5 hours prior to exercise were classified as “non-responders” as they did not demonstrate a marked increase in [NO_2_^-^]^28^. When those 10 participants consumed 8 mmol and 10 mmol, however, the number of “non-responders” were reduced to 2 and 1, respectively. As is evident in Figure 2A and 2B, our results yielded large standard deviations during the NR supplementation trials; this lends support to the possibility that the supplement dosage administered in this study was too low for some in our sample, although ≤6.2 mmol has been used by others in as little as 2.5 hours prior to exercise^7^^,^^29^^,^^30^. Therefore, a number of factors should be considered when determining the potential benefit of dietary NO_3_^-^ supplementation. The amount of dietary NO_3_^-^ consumed in this study may impact benefits and doses larger than 6.2 mmol may elicit different outcomes. As determined in the current study, aerobic fitness may be another factor which may alter supplement outcomes. Our low fit participants experienced a reduction in oxygen cost during low to moderate intensity exercise (≤60% VO_2max_) that was not found in high fit participants, consistent with other publications as previously described.

While the focus of this investigation was on the changes in oxygen cost during submaximal exercise, we also evaluated indicators of exertion including HR responses during each of the exercise bouts. A unique feature of our study was the inclusion of RPE during each of the five submaximal treadmill exercise bouts. While some elite and other aerobically trained participants may experience benefits from dietary NO_3_^-^ ^31^; it would appear that the current trend is that less benefit is received by elite and highly trained participants as compared to those with lower aerobic capacities^15^^,^^16^^,^^20^.

Dietary nitrate may increase bioavailability of NO and therefore may also be responsible for a number of changes including the following: increased blood flow via vascular smooth muscle relaxation and cyclic GMP accumulation, improved or increased exercise induced glucose uptake in skeletal muscle, neurotransmission, immune response, regulation of mitochondrial respiration, and glycolysis as mediated by AMPK^9^^,^^10^. Dietary NO_3_^-^ has been shown to improve endothelial function in healthy individuals with a lesser effect in older individuals (>60 years of age) as well as those with cardiovascular disease risk factors^32^. Future research examining the effect of dietary nitrate on endothelial function and arterial stiffness controlling for aerobic fitness may better explain the mechanisms for dietary nitrate and the cost of oxygen during submaximal exercise. Other areas which may also benefit from further research include the augmentation of performance of events using different muscle fiber types (type I vs. type II), as well as diseased and aging populations.

A greater benefit from NO_3_^-^ may, therefore, be observed in those participants who are less fit and have not received natural physiologic adaptation from aerobic training. The implications of our findings suggest that low fit men (VO_2max_ lower than 65% of their age-specific normative values) may experience greater reduction in submaximal oxygen consumption following NO_3_^-^ supplementation compared to individuals who have a higher fitness level (VO_2max_ greater than 85% of their age-specific normative values).

In summary, our findings support conclusions of others which have also reported that high fit individuals may receive less benefit than low fit individuals who consume dietary NO_3_^-^ supplements prior to physical activity^33^. In a commentary on this topic, Porcelli also reported on the effects of nitrate supplementation on oxygen consumption with consideration of aerobic fitness level. Our findings support Porcelli’s conclusions that the effects of nitrate supplementation are in fact significantly related to individual aerobic fitness levels, “with no benefits observed on highly trained subjects (VO_2max_ >60 ml/kg/min).” While more evidence is necessary, the same findings may also be true at higher elevations where high fit cyclists and runners alike did not receive exercise benefits following dietary NO_3_^-^ supplementation^34^^,^^35^.

Men with VO_2max_ lower than 65% of their age-specific normative values appear to be more likely to incur reduced oxygen consumption following dietary NO_3_^-^ supplementation during low to moderate intensity exercise (below 60% VO_2max_). Further, the greater benefit from dietary NO_3_^-^ supplementation may occur in individuals who have not received natural physiologic adaptation from aerobic training. Men with a VO_2max_ greater than 85% of their age-specific normative values may incur lesser reduction in submaximal oxygen cost following dietary nitrate supplementation when compared to low fit individuals.

## References

[1] Bailey SJ, Winyard P, Vanhatalo A, Blackwell JR, Dimenna FJ, Wilkerson DP, Tarr J, Benjamin N, Jones AM (2009). Dietary nitrate supplementation reduces the O2 cost of low-intensity exercise and enhances tolerance to high-intensity exercise in humans. *J Appl Physiol*..

[2] Lansley KE, Winyard PG, Fulford J, Vanhatalo A, Bailey SJ, Blackwell JR, DiMenna FJ, Gilchrist M, Benjamin N, Jones AM (2011). Dietary nitrate supplementation reduces the O2 cost of walking and running: a placebo-controlled study. *J Appl Physiol*..

[3] Larsen FJ, Weitzberg E, Lundberg JO, Ekblom B. (2007). Effects of dietary nitrate on oxygen cost during exercise. *Acta Physiol Oxf Engl*..

[4] Hord NG, Tang Y, Bryan NS. (2009). Food sources of nitrates and nitrites: the physiologic context for potential health benefits. *Am J Clin Nutr*..

[5] Larsen FJ, Weitzberg E, Lundberg JO, Ekblom B. (2010). Dietary nitrate reduces maximal oxygen consumption while maintaining work performance in maximal exercise. *Free Radic Biol Med*..

[6] Vanhatalo A, Bailey SJ, Blackwell JR, DiMenna FJ, Pavey TG, Wilkerson DP, Benjamin N, Winyard PG, Jones AM (2010). Acute and chronic effects of dietary nitrate supplementation on blood pressure and the physiological responses to moderate-intensity and incremental exercise. *Am J Physiol Regul Integr Comp Physiol*..

[7] Lansley KE, Winyard PG, Bailey SJ, Vanhatalo A, Wilkerson DP, Blackwell JR, Gilchrist M, Benjamin N, Jones AM (2011). Acute dietary nitrate supplementation improves cycling time trial performance. *Med Sci Sports Exerc*..

[8] Bescós R, Rodríguez FA, Iglesias X, Ferrer MD, Iborra E, Pons A. (2011). Acute administration of inorganic nitrate reduces VO(2peak) in endurance athletes. *Med Sci Sports Exerc*..

[9] Bailey SJ, Vanhatalo A, Winyard PG, Jones AM. (2012). The nitrate-nitrite- nitric oxide pathway: Its role in human exercise physiology. *Eur J Sport Sci*..

[10] Carriker C, Gibson A, Mermier C. (2013). The role of the nitrate-nitrite- nitric oxide pathway during hypoxia.

[11] Bailey SJ, Fulford J, Vanhatalo A, Winyard PG, Blackwell JR, DiMeFnna FJ, Wilkerson DP, Benjamin N, Jones AM (2010). Dietary nitrate supplementation enhances muscle contractile efficiency during knee-extensor exercise in humans.

[12] Boorsma RK, Whitfield J, Spriet LL. (2014). Beetroot juice supplementation does not improve performance of elite 1500-m runners. *Med Sci Sports Exerc*..

[13] Christensen PM, Nyberg M, Bangsbo J. (2013). Influence of nitrate supplementation on VO_2_ kinetics and endurance of elite cyclists.

[14] Peacock O, Tjønna AE, James P, Wisløff U, Welde B, Böhlke N, Smith A, Stokes K, Cook C, Sandbakk O (2012). Dietary nitrate does not enhance running performance in elite cross-country skiers. *Med Sci Sports Exerc*..

[15] Hoon MW, Johnson NA, Chapman PG, Burke LM. (2013). The effect of nitrate supplementation on exercise performance in healthy individuals: a systematic review and meta-analysis. *Int J Sport Nutr Exerc Metab*..

[16] Jonvik KL, Nyakayiru J, van Loon LJC, Verdijk LB. (2015). Can elite athletes benefit from dietary nitrate supplementation?.

[17] Thompson W, Gordon N, Pescatello L. (2010). ACSM’s Guidelines for Exercise Testing and Prescription.

[18] Borg G. (1970). Perceived exertion as an indicator of somatic stress. *Scand J Rehabil Med*..

[19] Govoni M, Jansson EA, Weitzberg E, Lundberg JO. (2008). The increase in plasma nitrite after a dietary nitrate load is markedly attenuated by an antibacterial mouthwash. *Nitric Oxide Biol Chem Off J Nitric Oxide Soc*..

[20] Porcelli S, Ramaglia M, Bellistri G, Pavei G, Pugliese L, Montorsi M, Rasica L, Marzorati M (2014). Aerobic Fitness Affects the Exercise Performance Responses to Nitrate Supplementation. *Med Sci Sports Exerc*..

[21] Pawlak-Chaouch M, Boissière J, Gamelin FX, Cuvelier G, Berthoin S, Aucouturier J. (2016). Effect of dietary nitrate supplementation on metabolic rate during rest and exercise in human: A systematic review and a meta-analysis. *Nitric Oxide Biol Chem*..

[22] Muggeridge DJ, Howe CCF, Spendiff O, Pedlar C, James PE, Easton C. (2014). A single dose of beetroot juice enhances cycling performance in simulated altitude. *Med Sci Sports Exerc*..

[23] Cermak NM, Gibala MJ, van Loon LJC. (2012). Nitrate supplementation’s improvement of 10-km time-trial performance in trained cyclists. *Int J Sport Nutr Exerc Metab*..

[24] Murphy M, Eliot K, Heuertz RM, Weiss E. (2012). Whole beetroot consumption acutely improves running performance. *J Acad Nutr Diet*..

[25] Larsen FJ, Schiffer TA, Borniquel S, Sahlin K, Ekblom B, Lundberg JO, Weitzberg E (2011). Dietary inorganic nitrate improves mitochondrial efficiency in humans. *Cell Metab*..

[26] Nair KS, Irving BA, Lanza IR. (2011). Can dietary nitrates enhance the efficiency of mitochondria?. *Cell Metab*..

[27] Vaughan RA, Gannon NP, Carriker CR. (2015). Nitrate-containing beetroot enhances myocyte metabolism and mitochondrial content. *J Tradit Complement Med*..

[28] Wylie LJ, Kelly J, Bailey SJ, Blackwell JR, Skiba PF, Winyard PG, Jeukendrup AE, Vanhatalo A, Jones AM (2013). Beetroot juice and exercise: pharmacodynamic and dose-response relationships. *J Appl Physiol*..

[29] Engan HK, Jones AM, Ehrenberg F, Schagatay E. (2012). Acute dietary nitrate supplementation improves dry static apnea performance. *Respir Physiol Neurobiol*..

[30] Wilkerson DP, Hayward GM, Bailey SJ, Vanhatalo A, Blackwell JR, Jones AM. (2012). Influence of acute dietary nitrate supplementation on 50 mile time trial performance in well-trained cyclists. *Eur J Appl Physiol*..

[31] Peeling P, Cox GR, Bullock N, Burke LM. (2014). Beetroot Juice Improves on-Water 500 m Time-Trial Performance, and Laboratory- Based Paddling Economy in National and International- Level Kayak Athletes. *Int J Sport Nutr Exerc Metab*..

[32] Lara J, Ashor AW, Oggioni C, Ahluwalia A, Mathers JC, Siervo M. (2016). Effects of inorganic nitrate and beetroot supplementation on endothelial function: a systematic review and meta‑analysis. *Eur J Nutr*..

[33] Hultström M, Amorim de Paula C, Antônio Peliky Fontes M, Porcelli S, Bellistri G, Pugliese L,  Rasica L,  Marzorati M, Pavei G, Ferguson SK, Holdsworth CT, Musch TI, Poole DC, Bourdillon N, Hoon MW, Burke LM, Michielli DW, Faiss R, Millet GP, Corona BT, Green MS, da Silveira AL, Sindler AL, Casey DP, Johnson BD, Wheatley CM, Carlson-Phillips A, Kunces LJ, Bescos R, Johnson LC, Martens CR, Justice JN, Ballak SB, Ballak DB, Wanner SP, Coimbra CC, Rehman S (2015). Commentaries on Viewpoint: Can elite athletes benefit from dietary nitrate supplementation?.

[34] Carriker CR, Mermier CM, Van Dusseldorp TA, Johnson KE, Beltz NM, Vaughan RA, McCormick JJ, Cole NH, Witt CC, Gibson AL (2016). Effect of Acute Dietary Nitrate Consumption on Oxygen Consumption During Submaximal Exercise in Hypobaric Hypoxia. *Int J Sport Nutr Exerc Metab*..

[35] Arnold JT, Oliver SJ, Lewis-Jones TM, Wylie LJ (2015). Macdonald JH. Beetroot juice does not enhance altitude running performance in well-trained athletes.

